# Datasets Construction and Development of QSAR Models for Predicting Micronucleus In Vitro and In Vivo Assay Outcomes

**DOI:** 10.3390/toxics11090785

**Published:** 2023-09-15

**Authors:** Lusine Khondkaryan, Ani Tevosyan, Hayk Navasardyan, Hrant Khachatrian, Gohar Tadevosyan, Lilit Apresyan, Gayane Chilingaryan, Zaven Navoyan, Helga Stopper, Nelly Babayan

**Affiliations:** 1 Institute of Molecular Biology, NAS RA, Yerevan 0014, Armenia; l_khondkaryan@mb.sci.am (L.K.); g_tadevosyan@mb.sci.am (G.T.); l_apresyan@mb.sci.am (L.A.); 2 Toxometris.ai, Yerevan 0009, Armenia; atevosyan@toxometris.ai (A.T.); hnavasardyan@toxometris.ai (H.N.); znavoyan@toxometris.ai (Z.N.); 3 YerevaNN, Yerevan 0025, Armenia; hrant@yerevann.com (H.K.); gayane@yerevann.com (G.C.); 4 Department of Informatics and Applied Mathematics, Yerevan State University, Yerevan 0025, Armenia; 5 Institute of Pharmacology and Toxicology, University of Würzburg, 97078 Würzburg, Germany; stopper@toxi.uni-wuerzburg.de

**Keywords:** micronucleus, in vitro, in vivo, prediction, ensemble, chemotypes analysis

## Abstract

In silico (quantitative) structure–activity relationship modeling is an approach that provides a fast and cost-effective alternative to assess the genotoxic potential of chemicals. However, one of the limiting factors for model development is the availability of consolidated experimental datasets. In the present study, we collected experimental data on micronuclei in vitro and in vivo, utilizing databases and conducting a PubMed search, aided by text mining using the BioBERT large language model. Chemotype enrichment analysis on the updated datasets was performed to identify enriched substructures. Additionally, chemotypes common for both endpoints were found. Five machine learning models in combination with molecular descriptors, twelve fingerprints and two data balancing techniques were applied to construct individual models. The best-performing individual models were selected for the ensemble construction. The curated final dataset consists of 981 chemicals for micronuclei in vitro and 1309 for mouse micronuclei in vivo, respectively. Out of 18 chemotypes enriched in micronuclei in vitro, only 7 were found to be relevant for in vivo prediction. The ensemble model exhibited high accuracy and sensitivity when applied to an external test set of in vitro data. A good balanced predictive performance was also achieved for the micronucleus in vivo endpoint.

## 1. Introduction

Evaluation of genotoxicity represents an integral part of the authorization of any industrial or pharmaceutical substance due to the association with severe health hazards, including cancer. A standard test battery is required by regulatory bodies for comprehensive assessment of major genotoxicity endpoints, covering gene mutation and structural (clastogenicity) and numerical (aneuploidy) chromosome damage [1]. The common strategy for genotoxicity testing, with slight modifications among various industrial sectors, includes in vitro mutagenicity testing by the reverse gene mutation (Ames) test, while chromosome damage is usually evaluated by in vitro micronucleus (MN) or chromosomal aberration (CA) assays, followed by in vivo tests. The choice of in vivo test largely depends on the range of genotoxic events detected in the in vitro studies [2]. Thus, a positive in vitro MN test is commonly followed by an in vivo MN assay.

The increasing number of chemicals under development represents a challenging task for regulatory agencies as a significant backlog of chemical substances that have either not undergone genotoxicity evaluation or have received insufficient assessment has appeared [3,4]. On the other hand, developers of any industrial chemical are deeply interested in assessing the genotoxic potential of new candidates before investing significant resources.

Thus, there is an urgent need for alternative high-throughput genotoxicity assessment methods. One such approach is in silico (quantitative) structure–activity relationship ((Q)SAR) modeling [5]. (Q)SAR models aim to find the relationships between chemical structural features and biological activity [6]. The cost-effective and time-saving nature of (Q)SAR approaches, along with their ability to address the concerns associated with the 3 Rs (replacement, refinement and reduction) principles of animal use, provide advantages over conventional testing methods. These characteristics make the in silico approach a valuable tool in early phases of product development, particularly for screening purposes. In recent years, (Q)SAR models have also been gaining importance in the regulatory frameworks [7,8,9]. The development of (Q)SAR models for genotoxicity prediction has been boosted with acceptance of the ICH M7 guideline, which focuses on evaluating and managing DNA reactive (mutagenic) impurities in pharmaceuticals and accepts in silico models for their evaluation [7]. During recent years, various models both commercially and publicly available for the prediction of the reverse gene mutation (Ames) test have been developed. The performance of these models on average reaches 80% accuracy, which is close to the reported inter-laboratory variation [5,10]. In contrast, models for predicting other genotoxicity endpoints, such as chromosome damage, are relatively scarce and less reliable [11]. One of the limiting factors appears to be the availability and quality of experimental test results databases [10,11]. Another constraining element is the models’ ability to handle imbalanced data, which is a very common problem in biomedical datasets, including genotoxicity data. In machine learning, imbalanced data represents a significant challenge, leading to a bias in a model’s predictive performance towards a majority class [12]. Thus, a classifier would have a good ability to predict samples that make up a large proportion of the data but perform poorly in predicting the minority. The selection of the algorithm and/or model architecture which is best suited for a particular task also presents a significant challenge. Moreover, (Q)SAR models should be constantly updated with new data to ensure broad chemical coverage, because models developed on small datasets have low predictive ability for new compounds.

Taking these into account, in the present study:

- We constructed a database for both in vitro and in vivo MN assays. This was achieved by searching through 35 million PubMed abstracts and extracting relevant data using the BioBERT pretrained large language model, which is designed for biomedical text mining [13]. The extracted data was subsequently reviewed and normalized by human experts.
- Chemotypes enrichment analysis was performed to identify substructures enriched in both datasets.
- Conventional and cutting-edge individual QSAR models were constructed based on consolidated datasets.
- Finally, an ensemble model was developed by combining these individual models.

## 2. Materials and Methods

### 2.1. Data Collection and Curation

In the present study two approaches were adopted for in vitro and in vivo MN dataset collection. First, data were retrieved from non-proprietary, publicly available databases which included:

- ISSMIC database on in vivo MN test results, which includes Toxnet, the National Toxicology Program and the Leadscope FDA CRADA Toxicity Database [6,14];
- EURL ECVAM Genotoxicity and Carcinogenicity dataset of Ames positive and Ames negative chemicals, which includes data on both in vitro and in vivo MN compiled from various sources [15,16];
- Chemical Carcinogenesis Research Information System [17];
- CHEMBL database (version 29), which contains data on chemical compounds’ structure and bioactivity extracted mainly from scientific literature [18].

Next, to extract data from publicly available literature we employed a pipeline based on the BioBERT model [13]. BioBERT (Bidirectional Encoder Representations from Transformers for Biomedical Text Mining) is a state-of-the-art biomedical language representation model based on BERT architecture and pretrained on large-scale biomedical corpora. In the present study we used BioBERT-Base v1.0 (+PubMed 200K) with the named entity recognition (NER) mode freely available at https://github.com/dmis-lab/biobert (accessed on 5 December 2022). Since BioBERT fine-tuning requires the availability of annotated task-specific corpora, we first downloaded the relevant titles and abstracts from Pubmed using simple keywords, such as, “in vitro”, “in vivo”, “micronucleus”, “micronuclei”. This resulted in 20,000 abstracts, out of which 2000 were manually annotated by four annotators. Controversial cases were verified by the domain expert. The collected and annotated data were used to fine-tune the BioBERT [13], using default parameters. Transformers library [19] on top of Pytorch [20] framework was used. The subsequent results were reviewed by domain experts and data on experimental results and compounds used were extracted from the publications. At the same time, studies were reviewed for their compliance with the OECD 487 [21] MN in vitro and 474 [22] MN in vivo test guidelines, respectively. Equivocal or technically compromised studies were removed from the datasets. Specifically, for MN in vitro database only, experiments conducted on human peripheral blood lymphocytes, CHO, V79, CHL/IU, L5178Y, TK6, HT29, Caco-2, HepaRG, HepG2, A549 and primary Syrian Hamster Embryo cells were included, taking into account the use of rat liver extract (S9) for negative results. As for in vivo MN, database results on bone marrow and/or blood erythrocytes were selected considering the highest tested dose and duration of treatment. Additionally, only studies reporting a statistically significant increase in micronucleated cells in one or more experimental groups were included as positive results. In cases where conflicting records existed for the same compound, the compound was either excluded from the final dataset, or the record that complied with the current regulatory criteria was retained. Two separate datasets for experimental results performed on mice and rats were constructed. To obtain SMILES of the tested chemicals, PubChem querying was performed based on the CAS numbers and/or name provided in the original source. Data were further cleaned to remove mixtures, polymers and inorganic and organometallic compounds, and by neutralization of salts. Finally duplicates from all datasets were removed by InChiKeys comparisons and Canonical Smiles were generated using RDKit package [23].

The curated final dataset consists of 894 organic chemicals with binary (positive/negative) MN in vitro experimental data, containing 70% positive and 30% negative compounds. Accordingly, the mouse MN in vivo database includes 1222 chemicals with 32% positive and 68% negative experimental data. Additionally, a set of 87 chemicals with MN in vitro and 87 with MN in vivo results was obtained from Baderna et al. [24] and Morita et al. [25], which was used as an external test set (see Section 2.6). The names, SMILES and CAS numbers of chemicals are provided in Tables S1 and S2 for MN in vitro and in vivo, respectively.

### 2.2. Structural Features Analysis by Chemotypes

To identify chemical substructures (i.e., chemotypes) that differentiate negative and positive chemicals in the target dataset and compare chemical spaces, Toxprint chemotypes were generated using freely available ChemoTyper application version 1.0 (https://chemotyper.org/, accessed on 12 May 2023). In total, 729 chemotypes were developed by Molecular Networks GmbH and Altamira, LLC for US Food and Drug Administration Center for Food Safety and Applied Nutrition and Office of Food Additive Safety based on different toxicity databases [26]. The ToxPrint chemotype enrichment analysis workflow (CTEW) described previously by Wang et al. [27] was applied. Based on a binary CT fingerprint table, a confusion matrix was generated, where true positives (TP) were defined as chemicals that contained the chemotype (CT) and had a positive label; true negatives (TN) were compounds that both had a CT negative label; false positives (FP) had a negative label but contained the CT; and false negatives (FN) did not have the CT but had a positive label. ODDs ratio was calculated using the following formula:

$$ODDs=\left(TP*TN\right)/(FN*FP)$$

One sided Fisher’s exact test was performed to evaluate significance of each CT and CTs were filtered based on the thresholds: ODDs ≥ 3 and *p* value < 0.5. Additionally, balanced accuracy (BA) for each CT and the full set of enriched CTs and Positive predictivity value (PPV) for each CT was calculated by:

BA=(SE+SP)/2

PPV=(TP+TN)/TP

### 2.3. Descriptors Calculation and Selection

For each of the datasets, 1D and 2D molecular descriptors were generated using the RDkit package [23]. In total, 208 descriptors were calculated, consisting mostly of physico-chemical properties and fraction of a substructure. Highly intercorrelated (R^2^ > 0.9), constant and low variance (std < 0.5) descriptors were removed at the preprocessing step. Finally, the optimal subset for each target dataset was determined using Genetic Algorithm [28]. In all, 12 types of molecular fingerprints, namely Toxprint, MACCS, Daylight and ECFP2, FCFP4 and ECFP6 with various lengths were calculated. Toxprint fingerprints were generated using Chemotyper application version 1 (https://chemotyper.org/, accessed on 12 May 2023) based on Toxprint chemotypes, while the rest was calculated using RDkit.

### 2.4. Data Balancing

To address for data imbalance, class weighting [29] and/or Synthetic Minority Over-sampling Technique (SMOTE) [30,31] was performed on the training set with ten-fold cross-validation, using a ratio of samples in the minority class with respect to the majority class corresponding to that of the training set. Class weighting allows for assigning weights to each class during the training step resulting in a balanced contribution of each one. The same balancing strategy was also applied for GCN using the Balancing Transformer as implemented in DeepChem library [32]. The idea behind the SMOTE technique is to create new synthetic data similar to existing samples in the minority class by finding their k nearest neighbors. For comparison, models trained without balancing were benchmarked against the same models trained using class weighting and SMOTE.

### 2.5. Model Development

In the present study, five ML models, namely random forest (RF) [33], Support Vector Machine (SVM) [34], eXtreme Gradient Boosting (XGB) [35], Graph Convolutional Networks [36] (GCN) and BARTSmiles [37] were evaluated. The first three are conventional ML algorithms applied on descriptors and fingerprints. GCN is a type of neural network that operates directly on graph-structured data, while recently proposed BARTSmiles represents a large language model based on BART-like architecture, that has demonstrated competitive results with the state-of-the-art self-supervised models in a wide range of chemical and biological tasks [37]. The BARTSmiles model is publicly available at https://github.com/YerevaNN/BARTSmiles/ (accessed on 16 June 2023). The hyperparameters optimization for RF, SVM and XGB models was carried out on the training set using a grid search in an inner ten-fold cross-validation with the scikit-learn library for Python [38]. To reduce computational cost, GCN and BARTSmiles were optimized with respect to their hyperparameters using Butina split as implemented in RDkit [39].

The best-performing models were used to build an ensemble classifier. As has previously been shown, ensemble methods, which combine multiple individual models via voting or averaging, in general show better predictive performance than individual ones [40].

### 2.6. Model Performance Evaluation

All models were evaluated using a ten-fold cross-validation by splitting the data into 90% training and 10% validation sets using Stratified shuffle split of scikit-learn [38]. Additionally, models were evaluated on the external test set (see Section 2.1). For evaluating the performance of the models, the following metrics were used: the area under the curve (AUC), accuracy (Acc), sensitivity (SE) and specificity (SP). All metrics were calculated based on the confusion matrices created from the number of true-positive (TP), true-negative (TN), false-positive (FP) and false-negative (FN) predictions using the following formulas:

$$Acc=\frac{\left(TP+TN\right)}{\left(TP+TN+FP+FN\right)}$$

$$SE=\frac{TP}{\left(TP+FN\right)}$$

$$SP=\frac{TN}{\left(TN+FP\right)}$$

where Acc displays the ability of the model to correctly predict all positive samples as positive ones; SE reflects the potential of the model to correctly classify a sample as positive, while SP is the ability to correctly classify a sample as negative taking into account all positive or negative data points, respectively. The AUC is the measure of the predictive ability of a model. The higher the AUC, the better the classifier’s performance at differentiating between negative and positive classes.

The parameters were determined for each fold of validation, and average values of each scoring matrix, including Acc, SE, SP and AUC, were calculated to select the best model.

## 3. Results and Discussion

### 3.1. Datasets

Chemical libraries for Q(SAR) models should constantly be updated to ensure better predictive performance and high coverage. To the best of our knowledge, only recently was the first dataset on MN in vitro, consisting of 380 samples, reported by Baderna et al. [24]. By utilizing a cutting-edge text-mining technique, we managed to increase this number by almost three times. The mouse MN in vivo database increased by 308 chemicals compared to the lately published one by Yoo et al. [41].

**Figure 1:**
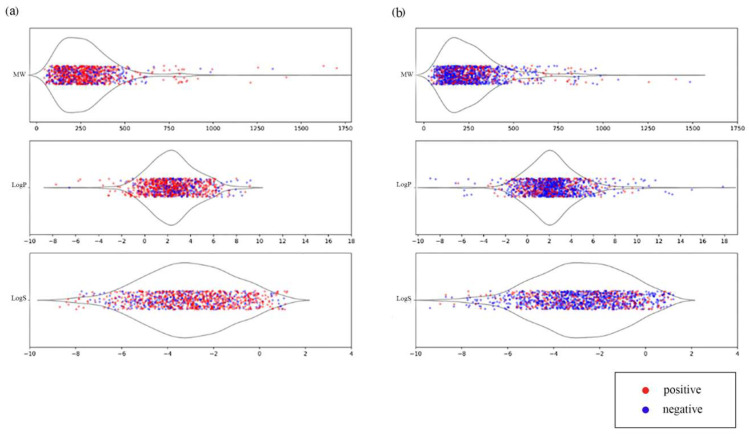
Distribution of physicochemical properties for MN in vitro (**a**) and MN in vivo (**b**) datasets. From top to bottom the following properties are presented: Molecular weight (MW), octanol–water partition coefficient (logP) and water solubility (logS). Dots are values of the property for each chemical, the violin plots represent the number of compounds with the same values (density). Positive compounds are colored in red and negatives in blue.

The distribution of the main physicochemical properties, namely molecular weight (MW), octanol–water partition coefficient (logP) and aqueous solubility (logS) of the chemicals in the final MN in vitro and MN in vivo databases, is shown in Figure 1. MW and logP was calculated using the RDkit package, while the ALOGPS software was used to compute logS [42]. As is evident from Figure 1, both datasets contain mostly small molecules (MW < 500), though a slightly higher number of heavier compounds with MW > 500 is found in in vivo data. The majority of chemicals in both datasets are characterized by logP values between −2 and 6 and logS above 10^−2^, which correlate with good bioavailability and solubility. Thus, there is no bias towards any specific type of chemicals with certain properties in both datasets.

**Figure 2:**
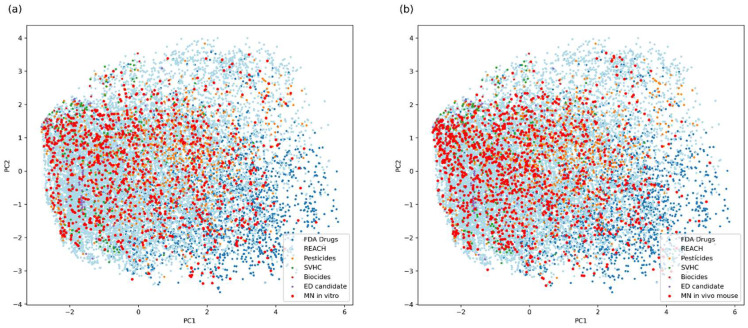
2D PCA visualization of chemical space of compounds found in MN in vitro (**a**) and in vivo (**b**) datasets and in the lists of REACH registered substances, FDA Drugs, pesticides, biocides, substances of very high concern (SVHC) and endocrine disruptor candidates (ED candidates). Data points represent compounds encoded as 166-bit MACCS fingerprints on the first two principal component dimensions.

For more detailed description of the chemicals in datasets, we compared the chemical space occupied by these compounds to the one covered by chemicals from databases, which include REACH registered substances, FDA drugs, pesticides, biocides, substances of very high concern (SVHC) and endocrine disruptor candidates (ED candidates) [43,44,45,46,47,48]. For comparison, Principal Component analysis (PCA) was performed based on MACCS fingerprints. It is worth mentioning that for some parts of these databases no structures could be retrieved; thus, the final number of chemicals in each dataset is as follows: REACH: n = 14,790; FDA Drugs: n = 3234; pesticides: n = 1028; biocides: n = 235; SVHC: n = 470; and ED candidates: n = 145. The results are shown in Figure 2, where structurally dissimilar chemicals are found far apart from each other. Both MN in vitro and in vivo datasets covered vast areas of the chemical space, indicating that the datasets contain highly diverse chemicals. The exceptions are the top-right and bottom-right areas, sparsely populated by substances from both datasets, which are primarily occupied by REACH chemicals and FDA Drugs.

**Figure 3:**
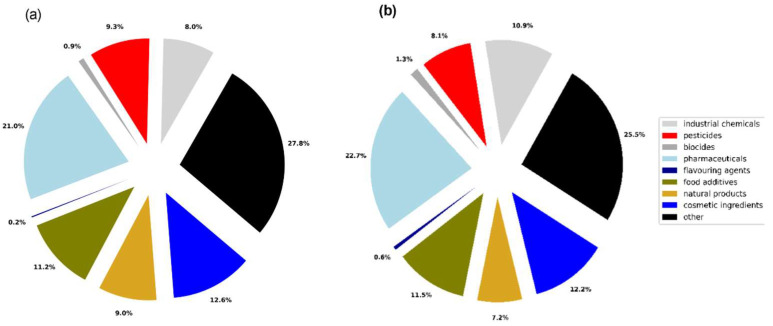
Product type categories within datasets (**a**) MN in vitro; (**b**) MN in vivo.

We also performed the evaluation of MN in vitro and in vivo substances by their main use and manufacturing using the PubChem database. The results are shown in Figure 3. The majority of substances in both datasets are represented by pharmaceuticals, followed by cosmetic ingredients and food additives.

### 3.2. Structural Feature Analysis by Chemotypes

To search for potential structure–activity associations, we applied chemotype enrichment analysis based on ToxPrint chemical features. Chemotype enrichment analysis results for MN in vitro yielded 18 positively enriched CTs. The full lists and statistics are provided in Table S3. Among the significantly positively enriched CTs were nitroso, steroids, alkyl halides and PAH-phenanthrene. In order to give a rough estimate of the coverage, 1 or more of the 18 positively enriched CTs was found in 263 compounds or only 39% of the MN in vitro positives. However, 95% of the 169 chemicals that contain 2 or more CTs were correctly predicted as MN positives. To evaluate a predictive performance of the full set of 18 positively enriched CTs, overall BA was calculated that reached 0.65, indicating overall a moderate predictive performance.

For MN in vivo, 40 positively enriched CTs (Table S4) were identified. CTs significantly enriched in a positive space included nitroso, metal and phosphorous substructures, usually found in environmental chemicals, and “ring:hetero_” CTs common for drug-like compounds. Despite the high number of positively enriched CTs, 1 or more of these CTs was found only in 37% of TPs (157 out of 426 chemicals), while 77% of chemicals that contain 2 or more CTs were correctly predicted as positives. Using all the CTs enriched in positive space, the overall BA of 0.64 was found, which indicates a moderately good predictive performance of the full set.

**Figure 4:**
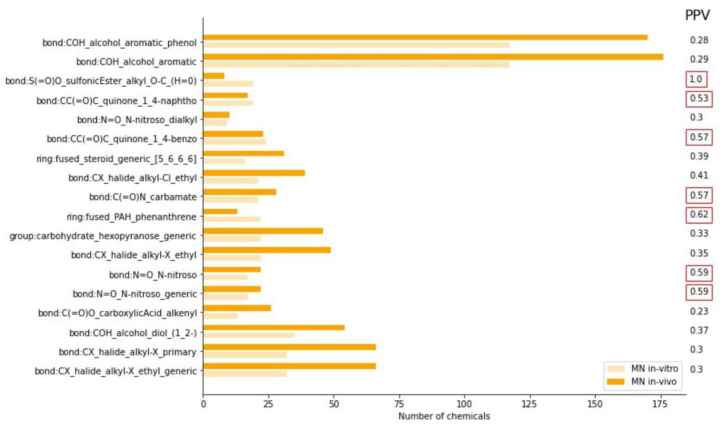
ToxPrint CTs enriched in the positive space of MN in vitro dataset (light orange) relative to the MN in vivo dataset (dark orange) with positive predictivity values (PPV) indicated on the right. PPV values ≥ 70% and 50% < PPV < 70% are enclosed in red boxes.

The positive CTs that are common for both endpoints represent a particular interest. Previously, based on expert assessment, Canipa et al. [49] reported 19 structural alerts that can predict both in vitro and in vivo chromosome damage without differentiating between chromosomal aberration and MN in vivo tests. In our study, we identified only 4 CTs enriched in the positive space of both datasets, particularly nitroso substructures, PAH_ phenanthrene and S(=O)O_sulfonicEster_alkyl_O-C_(H=0). To further explore the relevance of CTs over-represented in MN in vitro dataset for in vivo prediction, PPV for each CT enriched in MN in vitro was calculated for the MN in vivo dataset. CTs with PPV ≥ 70% were considered highly relevant for MN in vivo, while CTs with 50% < PPV < 70% and PPV < 50% were considered as moderately and poorly correlated with in vivo data, respectively (Figure 4). Among 18 CTs positively enriched in MN in vitro data, only 1 was found to be strongly associated with MN in vivo (PPV ≥ 70%), specifically “bond:S(=O)O_sulfonicEster_alkyl_O-C_(H=0)”. Alkyl esters of alkyl or sulfonic acids induce genotoxicity via DNA intercalating mechanism and present a significant safety challenge to drug producers and regulators [50]. Meanwhile, 6 CTs showed PPVs between 50% and 70%, indicating moderate relevance for in vivo prediction.

**Figure 5:**
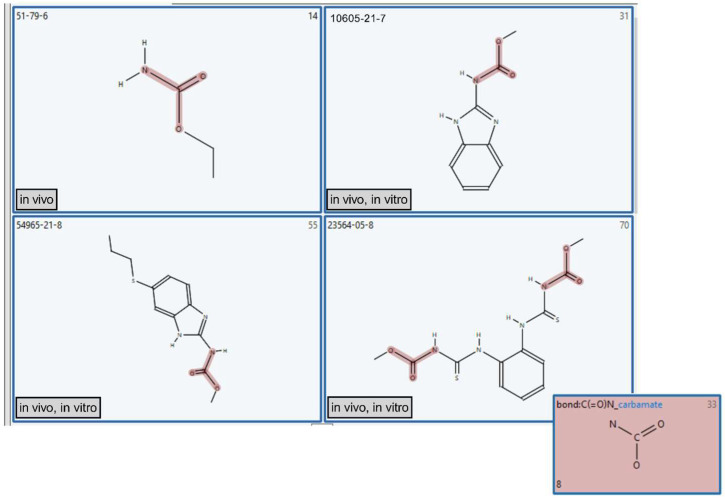
Representative images of chemicals containing “bond:C(=O)N_carbamate” CT (highlighted in red), labeled by CAS number and MN in vitro and in vivo activities.

For further illustration, we concentrated on the “bond:C(=O)N_carbamate” CT, which was found positively enriched in the MN in vitro dataset and moderately associated with in vivo activity (PPV < 70%). Figure 5 demonstrates images of four representative compounds with their indicated CAS numbers (CASN) and MN activity. Three out of four representative compounds induce MN both in vitro and in vivo, while urethane (CASN 51-79-6) has been reported to induce MN only in vivo. Urethane belongs to the carbamates chemical class and has been reported to induce MN in vivo but not in vitro. Though structural alerts for carbamate mutagenicity [51,52] have been reported, more recent thorough evaluation of this group revealed that only a small number of compounds, particularly urethane, demonstrate mutagenic activity in Ames tests via DNA adducts formation. Moreover, this effect is observed only when urethane is tested at very high concentrations (above limits for relatively non-toxic compounds). In contrast, it tests positive in an MN in vivo test. The most widely accepted explanation for this discrepancy is that urethane-associated DNA adducts are rather formed by its metabolite [53]. The S9 fraction used in an in vitro test is likely deficient in some cytochrome 450 enzymes responsible for urethane metabolism, while the DNA reactive metabolite is readily formed in vivo. Contrary to urethane, the other three chemicals, namely carbendazim (CASN 10605-21-7), albendazole (CASN 54965-21-8) and thiophanate-methyl (CASN 23564-05-8) have been reported to induce MN both in vitro and in vivo by directly interacting with tubulin and thus causing aneugenicity [54,55].

In summary, CT enrichment analysis revealed a range of substructures, such as nitroso, quinone, polycyclic hydrocarbons and aziridine, all of which have previously been identified as genotoxicity-related structural alerts [24]. In overall, the data mining approach employed in this study using ToxPrints CTs is chemically intuitive and straightforward to implement and interpret.

### 3.3. Selection of Data Balancing Method

In this study, to deal with highly imbalanced data, we tried two types of data balancing methods, namely class weights [29] and SMOTE [30,31], aiming to obtain a model that can consistently predict positive and negative samples with balanced SE and SP, while maintaining a high AUC value. It is worth mentioning that no balancing method is available for BARTSmiles.

To reduce the number of combinations and computational time, we assessed balancing strategies using the combination of RF with descriptors and MACCS fingerprints. The main reason for choosing the above-mentioned algorithm/fingerprint combination is that MACCS fingerprints and RF have been proven to be one of the most common and successful combinations in various fields of chemoinformatics over the years [56,57].

**Figure 6:**
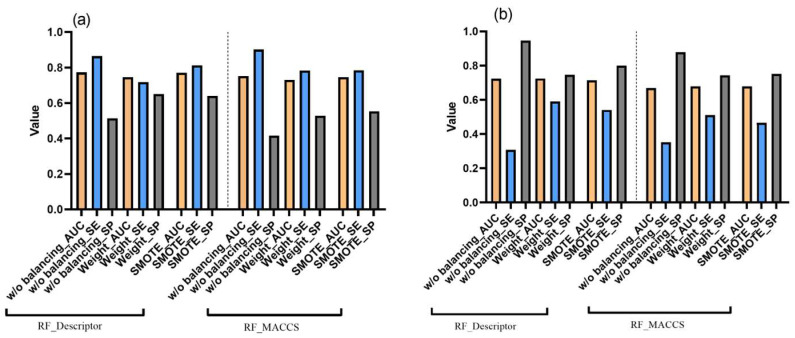
Performance of the RF models without balancing and using class weighting and SMOTE balancing methods in combination with molecular descriptors and MACCS fingerprints trained on the MN in vitro (**a**) and MN in vivo data (**b**), respectively. Average values of ten-fold cross-validation are presented.

As shown in Figure 6, both balancing strategies improved the model’s predictive balance for both datasets compared to the performance without balancing, despite similar AUC values. A comparison of strategies for MN in vitro data (Figure 6a) revealed that though SE and SP were comparable among the techniques, class weight balancing is characterized by a slightly lower AUC value (0.746 for descriptor- and 0.73 for fingerprint-based models, respectively) as opposed to SMOTE (0.77 for descriptor- and 0.75 for fingerprint-based models, respectively).

In contrast, training on the mouse MN in vivo data using SMOTE resulted in low SE (0.54 and 0.52 for descriptor- and fingerprint-based models, respectively) and high SP (0.8 and 0.81 for descriptor- and fingerprint-based models, respectively) (Figure 6b). At the same time, the class weight approach was found to give a more stable prediction accompanied by a higher AUC for the descriptor-based model. The detailed evaluation results are presented in Tables S5 and S6.

### 3.4. Selection of Molecular Fingerprints and Model Development

In the present study, we developed multiple models for each target endpoint using the combination of three classical ML algorithms (RF, SVM and XGB) with molecular descriptors and 12 types of fingerprints (MACCS, Daylight, Toxprint and ECPF with different bits) through ten-fold cross-validation. All models were trained using an appropriate balancing method.

Following feature selection (see Section 2.3), 17 and 20 molecular descriptors were used for building MN in vitro and in vivo models, respectively. The full list of descriptors is presented in Table S7. It is worth mentioning that for both endpoints the selected descriptors predominantly represent structural fragments rather than physico-chemical ones. Fingerprints were used without feature reduction. We selected the best performing combination based on the AUC values and balanced performance, ensuring an equal ability to predict both positive and negative classes. The performance of descriptor-based models for both datasets is presented in Table 1. The obtained results suggested that all models performed equally well with a slight superiority of the RF algorithm for MN in vitro and XGB for MN in vivo.

**Figure 7:**
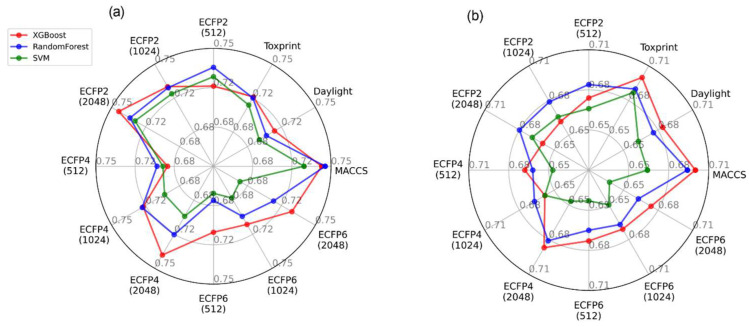
AUC values of models in combination with different fingerprints on the (**a**) MN in vitro and (**b**) MN in vivo, respectively. Models were trained using SMOTE or class weights balancing for MN in vitro and in vivo, respectively. Average values of ten-fold cross-validation are presented. Numeric values are presented in Tables S8 and S9.

The performance of various combinations of fingerprints/models is shown in Figure 7. All models demonstrated AUC values around 0.7 for both datasets and across all combinations, indicating good predictive ability. However, based on the most optimal parameters of internal validation (i.e., AUC/SE/SP) MACCS with RF was chosen as a final combination for MN in vitro endpoints, while Toxprint and MACCS fingerprints with XGB were selected for MN in vivo.

### 3.5. Model Validation

**Figure 8:**
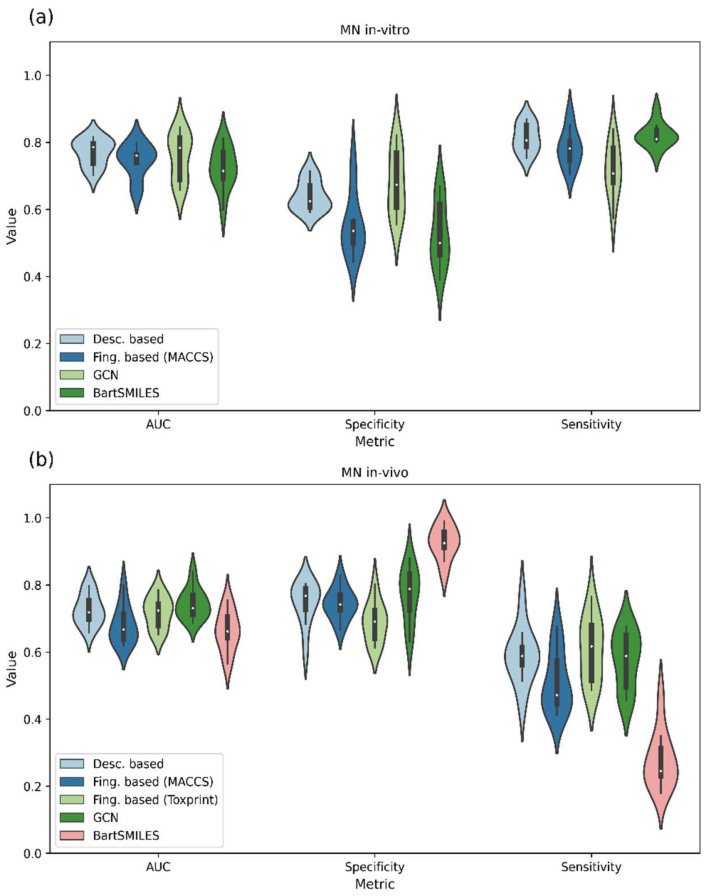
Distribution of AUC, Specificity and Sensitivity values obtained within the ten-fold cross-validation framework for (**a**) MN in vitro and (**b**) MN in vivo. Models were trained using SMOTE or class weights balancing for MN in vitro and in vivo, respectively. Average values of ten-fold cross-validation are presented.

Two conventional ML methods (RF and XGB) combined with selected molecular descriptors and fingerprints (MACCS and MACCS and Toxprint fingerprints for MN in vitro and MN in vivo, respectively) and two cutting-edge algorithms, namely GCN and BARTSmiles, were used for target endpoint prediction. The performance of the models obtained through a ten-fold cross-validation framework using balanced data where appropriate is presented in Figure 8. Among individual models, the best predictive performance for the MN in vitro dataset was achieved with RF in combination with descriptors using SMOTE balancing (0.77, 0.81 and 0.64 for AUC, SE and SP, respectively). In contrast, for MN in vivo, GCN showed a superior performance with AUC of 0.74, SE of 0.58 and SP of 0.77. It is worth mentioning that though both target datasets are highly imbalanced, BARTSmiles performed comparably to other models for the MN in vitro dataset in terms of AUC, SE and SP. However, for the MN in vivo dataset, its predictive ability is highly skewed towards the prediction of negative samples (SE of 0.23 and SP of 0.93).

To further assess the predictive power, the models were evaluated on the external test set. RF_Desc + SMOTE and RF_MACCS + SMOTE displayed equally good predictive potential on the MN in vitro dataset (Table 2a). On the MN in vivo external test set, most models showed a comparable prediction performance, with a slight predominance of the XGB model build using MACCS fingerprints (Table 2b).

To overcome the limitations of individual models, the ensemble model via majority voting was built. As expected, the ensemble model outperformed any single-base classifier, achieving higher Acc (78.4% and 73% for MN in vitro and in vivo data, respectively).

### 3.6. Comparison with Previous Models

Recently, Baderna et al. [24] reported a fragment-based model for MN in vitro prediction with Acc, SE and SP of 0.85, 0.98 and 0.62 in the validation set. Using the same set, which allowed us to directly compare the results, we achieved a lower prediction performance. Nonetheless, taking into account the high diversity of our dataset and the size of the training set, our model may have broader applicability and better predictivity for new compounds, which is highly practical for the early screening purposes of in silico models.

Conversely to MN in vitro, a number of in vivo prediction models exist [40,41,58,59]. Using commercial CASE Ultra software for MN in vivo prediction, Morita et al. [25] on the external dataset of 337 chemicals reported Acc, SE and SP of 0.72, 0.91 and 0.57. Though SE obtained in our study is lower, SP is particularly high. Moreover, the authors mention a possibility that the test and training set included the same chemicals, which is not the case in our study. More recently, Yoo et al. [41] developed a statistics-based model for the mouse dataset comprising 1001 compounds using Leadscope and CASE Ultra software. On the external test set of 42 compounds, the new models achieved SE of 67% and 83% and SP of 84% and 29% for Leadscope and CASE Ultra, respectively. Thus, compared to the models of Yoo et al. [41] our model reached balanced SE and SP, resulting in greater stability.

## 4. Conclusions

In this study, we first enriched the dataset for MN in vitro and mouse in vivo assays by leveraging freely available databases and conducting an extensive PubMed search, supported by the advanced text-mining approach based on the BioBERT large language model.

Using the updated datasets, we identified chemotypes, i.e., structural features associated with MN induction in vitro or in vivo. At the same, seven chemotypes that are positively enriched in the MN in vitro dataset and possess predictive value against MN in vivo were found. We constructed a number of individual models using conventional ML methods, such as RF, SVM and XGB, in combination with various fingerprints, molecular descriptors and balancing methods. Our findings from ten-fold cross-validation highlighted the superior performance of the MACCS fingerprint for MN in vitro prediction, while Toxprint and MACCS fingerprints excelled for MN in vivo prediction. Additionally, our analysis of various balancing techniques revealed that SMOTE for MN in vitro and class weights for MN in vivo achieved the optimal balance in terms of SE and SP in predictive performance. We also explored advanced modeling approaches, such as GCN and BARTSmiles, a large pre-trained generative masked language model. The performance of individual models on MN in vitro achieved accuracy values ranging from 66.7% to 75.9%, while for in vivo the accuracy values ranged from 56.3% to 65.5%. To further enhance predictive performance, ensemble models were constructed, resulting in an accuracy of 78.38% for MN in vitro and 71.26% for the in vivo dataset. In comparison to previous models, we successfully achieved a highly balanced classification for the latter endpoint.

## Figures and Tables

**Table 1:** Performance of models in combination with selected molecular descriptors on MN in vitro and MN in vivo datasets. Models were trained using SMOTE or class weights balancing for MN in vitro and in vivo, respectively. Average values of ten-fold cross-validation are presented. The best performing model is in bold.

Model	RDkit Molecular Descriptors (AUC/Sensitivity/Specificity)	
	MN In Vitro	MN In Vivo
XGB	0.74/0.756/0.65	**0.725/0.59/0.747**
RF	**0.776/0.81/0.644**	0.728/0.425/0.81
SVM	0.716/0.67/0.65	0.65/0.69/0.52

**Table 2:** Performance of individual and ensemble models on the MN (a) in vitro and (b) MN in vivo mouse external datasets. The best model in terms of Acc and balanced performance is in bold.

**(a)**				
**Model**	**Acc**	**SP**	**SE**	**AUC**
RF_Desc + SMOTE	72.41	0.548	0.821	0.76
RF_MACCS + SMOTE	75.86	0.613	0.839	0.747
GCN + class weight	67.816	0.645	0.696	0.740
BARTSmiles	66.666	0.516	0.750	0.675
Ensemble majority	**78.38**	**0.593**	**0.894**	-
**(b)**				
**Model**	**Acc**	**SP**	**SE**	**AUC**
RF_Desc + SMOTE	56.3	0.58	0.541	0.63
RF_MACCS + SMOTE	65.5	0.7	0.6	0.68
GCN + class weight	63.2	0.64	0.62	0.67
BARTSmiles	65.5	0.82	0.43	0.69
Ensemble majority	**71.26**	**0.72**	**0.703**	-

## Data Availability

The names, SMILES and CAS numbers of chemicals are available in Tables S1 and S2 for MN in vitro and in vivo, respectively. Toxicity data is available from the corresponding author upon reasonable request. The code for BARTSmiles is available at https://github.com/YerevaNN/BARTSmiles/ (accessed on 6 September 2023).

## Supplementary Materials

The following supporting information can be downloaded at: https://www.mdpi.com/article/10.3390/toxics11090785/s1, Table S1: The names, SMILES and CAS numbers of MN in vitro dataset, Table S2: The names, SMILES and CAS numbers of MN in vivo dataset, Table S3: CTs enriched in the positive space of MN in vitro, Table S4: CTs enriched in the positive space of MN in vivo, Table S5: Balancing strategy selection for MN in vitro, Table S6: Balancing strategy selection for MN in vivo, Table S7: Descriptors list, Table S8: Fingerprint/algorithm selection for MN in vitro; Table S9: Fingerprint/algorithm selection for MN in vivo.
