# Supplementary material for: Datasets Construction and Development of QSAR Models for Predicting Micronucleus In Vitro and In Vivo Assay Outcomes
Source: Toxics. 2023 Sep 15;11(9):785. doi: 10.3390/toxics11090785 (PMC10537630; doi:10.3390/toxics11090785)
Supplement: Supplementary file 1 [file toxics-11-00785-s001.zip › Supplementary_Table S5-S9.pdf]

Supplementary Information

Table S5. Balancing strategy selection for MN in vitro. The best values are in bold.

**Balancing strategy**	**Descriptors +RF**			**MACCS+RF**		
	AUC	Specificity	Sensitivity	AUC	Specificity	Sensitivity
SMOTE	**0.771**	0.64	**0.813**	**0.746**	**0.553**	**0.784**
Class weighting	0.746	**0.651**	0.718	0.731	0.528	0.783

Table S6. Balancing strategy selection for MN in vitro. The best values are in bold.

**Balancing strategy**	**Descriptors +RF**			**MACCS+RF**		
	AUC	Specificity	Sensitivity	AUC	Specificity	Sensitivity
SMOTE	0.715	**0.8**	0.541	0.679	0.752	0.466
Class weighting	**0.725**	0.747	**0.59**	**0.68**	**0.744**	**0.512**

Table S7. Descriptors list determined using Genetic Algorithm, after removing highly intercorrelated (R^2>0.9) and constant

**MN in vitro**	**MN in vivo**
MaxEStateIndex	PEOE_VSA6'
PEOE_VSA4	'SlogP_VSA3'
SMR_VSA10	'SlogP_VSA6'
SMR_VSA2	'EState_VSA8'
SMR_VSA4	NHOHCount
SlogP_VSA10	fr_Ar_COO
SlogP_VSA12	fr_Ar_NH
NumAliphaticRings	fr_NH0
fr_Ar_N	fr_SH
fr_Ndealkylation2	fr_amide
fr_SH	fr_aniline
fr_bicyclic	fr_benzene
fr_ester	fr_epoxide
fr_lactone	fr_hdrzine
fr_phenol_noOrthoHbond	fr_isocyan
fr_sulfone	fr_morpholine
fr_unbrch_alkane	fr_nitro
	fr_phenol_noOrthoHbond
	fr_sulfide
	fr_term_acetylene

Table S8. Fingerprint/algorithm selection for MN in vitro dataset. AUC/ Specificity/Sensitivity is indicated. The best values are in bold.

Fingerprint	RF	SVM	XGB
Maccs	**0.745/0.553/0.784**	**0.727/0.525/0.769**	**0.742/0.583/0.773**
Daylight	0.702/0.460/0.839	0.695/0.499/0.769	0.710/0.518/0.794
ECPF2 (512)	0.734/0.572/0.768	0.726/0.507/0.812	0.718/0.619/0.695
ECPF2 (1024)	0.727/0.558/0.753	0.721/0.493/0.815	0.728/0.644/0.693
ECPF2 (2048)	0.732/0.591/0.763	**0.727/0.493/0.813**	**0.743/0.622/0.708**
ECPF4 (512)	0.698/0.431/0.818	0.693/0.413/0.820	0.689/0.553/0.716
ECPF4 (1024)	0.720/0.482/0.799	0.698/0.435/0.825	0.719/0.611/0.724
ECPF4 (2048)	0.717/0.514/0.776	0.699/0.439/0.815	0.737/0.640/0.714
ECPF6 (512)	0.679/0.363/0.855	0.673/0.381/0.825	0.706/0.546/0.752
ECPF6 (1024)	0.699/0.414/0.823	0.681/0.403/0.825	0.707/0.590/0.718
ECPF6 (2048)	0.709/0.474/0.792	0.676/0.403/0.714	0.727/0.644/0.708
Toxprint	0.717/0.575/0.753	0.710/0.518/0.773	0.718/0.597/0.732

Table S9. Fingerprint/algorithm selection for mouse MN in vivo dataset. AUC/ Specificity/Sensitivity is indicated. The best values are in bold.

Fingerprint	RF	SVM	XGB
Maccs	**0.694/0.771/0.489**	**0.664/0.726/0.509**	**0.70/0.744/0.512**
Daylight	0.676/0.808/0.398	0.663/0.723/0.474	0.684/0.783/0.483
ECPF2 (512)	0.684/0.746/0.503	0.666/0.791/0.421	0.674/0.663/0.598
ECPF2 (1024)	0.679/0.732/0.512	0.666/0.796/0.416	0.662/0.658/0.604
ECPF2 (2048)	0.68/0.723/0.523	0.669/0.791/0.436	0.66/0.648/0.599
ECPF4 (512)	0.662/0.811/0.366	0.647/0.8/0.36	0.668/0.723/0.526
ECPF4 (1024)	0.667/0.775/0.439	0.658/0.809/0.381	0.658/0.666/0.579
ECPF4 (2048)	0.681/0.736/0.508	0.647/0.799/0.349	0.687/0.653/0.631
ECPF6 (512)	0.665/0.832/0.314	0.643/0.804/0.308	0.673/0.755/0.494
ECPF6 (1024)	0.667/0.799/0.364	0.65/0.819/0.308	0.671/0.708/0.532
ECPF6 (2048)	0.663/0.752/0.448	0.638/0.805/0.294	0.674/0.674/0.585
Toxprint	0.690/0.695/0.561	0.687/0.735/0.537	**0.70/0.689/0.60**7
